# Supplementary material for: Combined loss of LAP1B and LAP1C results in an early onset multisystemic nuclear envelopathy
Source: Nat Commun. 2019 Feb 5;10:605. doi: 10.1038/s41467-019-08493-7 (PMC6363790; doi:10.1038/s41467-019-08493-7)
Supplement: Supplementary file 1 — Supplementary Information [file 41467_2019_8493_MOESM1_ESM.pdf]

Combined loss of LAP1B and LAP1C results in an early onset multisystemic nuclear envelopathy

Fichtman et al.

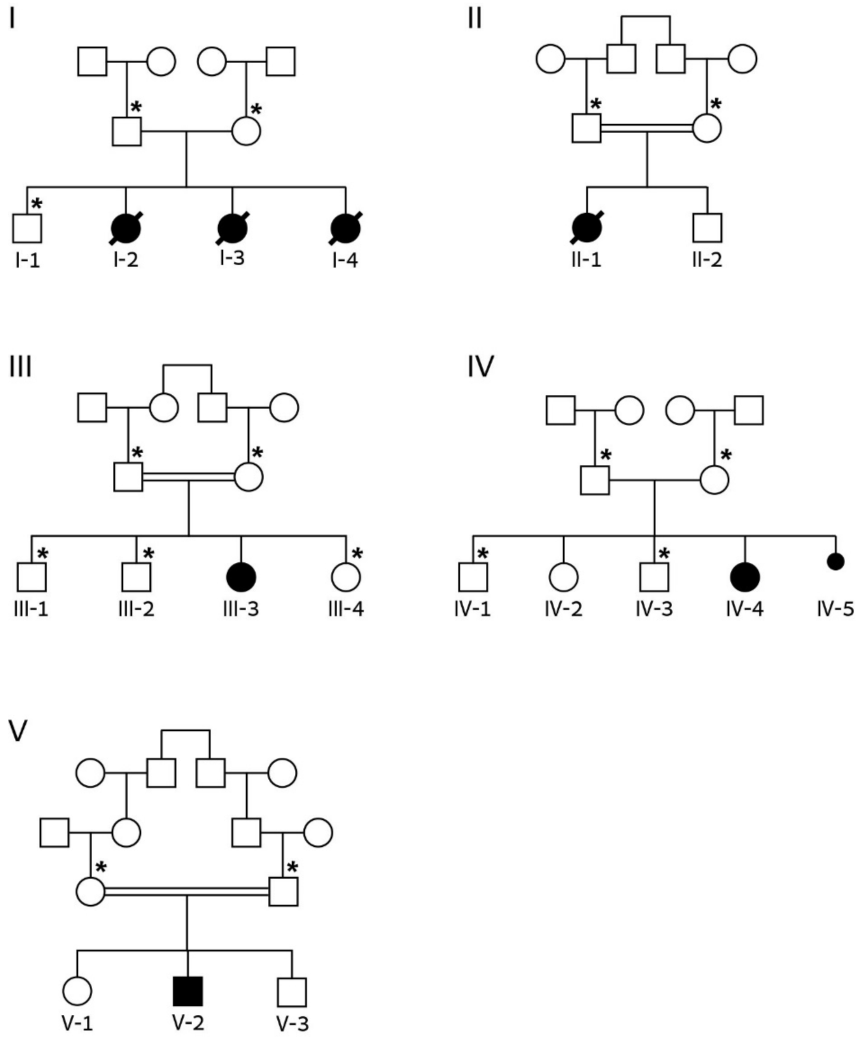

Supplementary Figure 1. Pedigrees of the affected families (I-V). Sanger sequencing was performed for all affected individuals, their parents and healthy siblings, except V-1 and V-3. Filled symbols indicate affected individuals; slanted bars indicate deceased; consanguineous relationships are depicted by a double horizontal line; asterisks indicate heterozygous individuals verified by sequencing. In family IV, prenatal diagnosis performed by chorionic villous sampling (CVS) identified an affected homozygous fetus, which permitted the early first trimester termination of pregnancy.

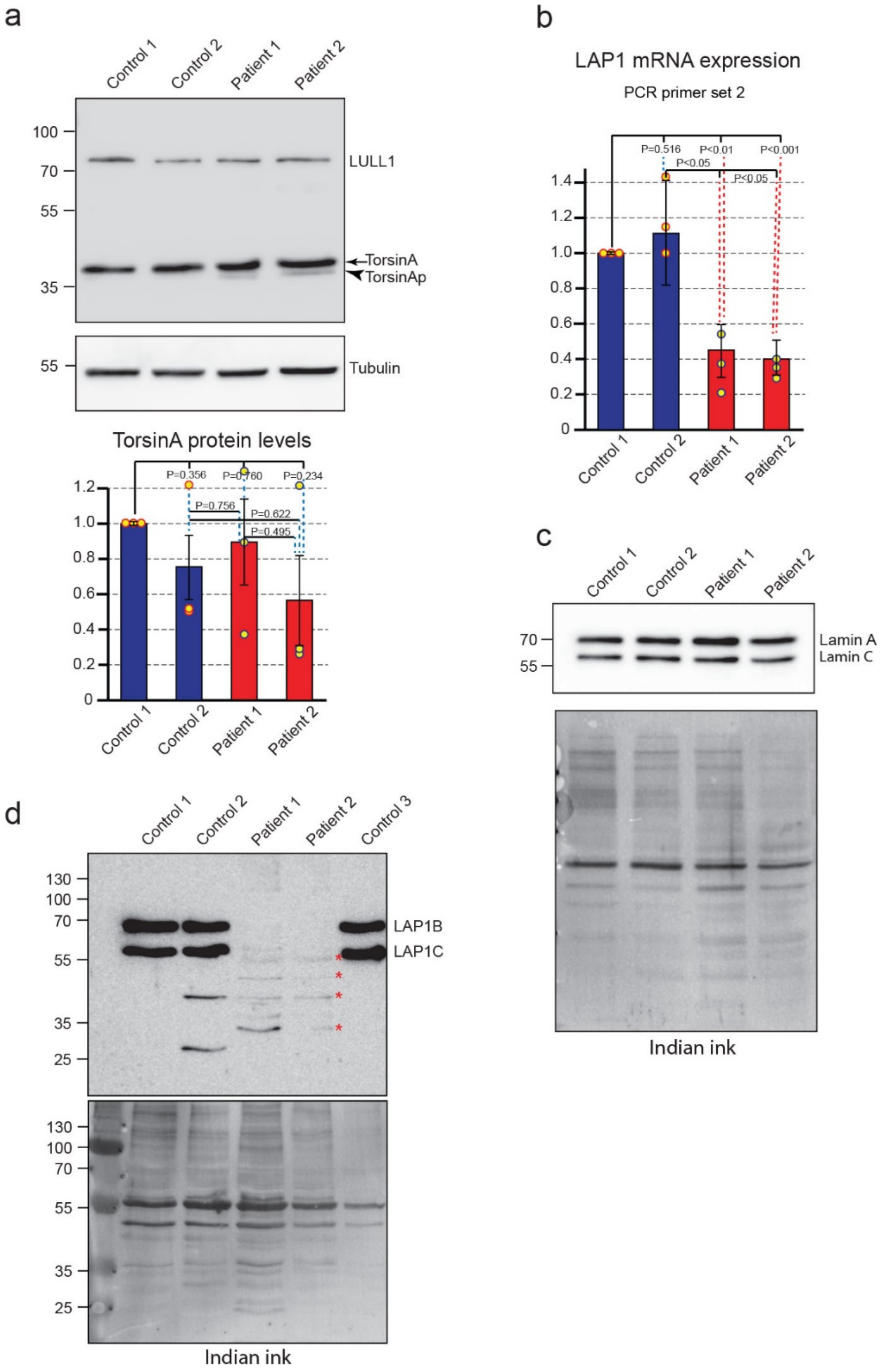

Supplementary Figure 2. Expression levels of LAP1 and its interactors. Western immunoblot analysis of cell lysates was performed as in Figure 2c and qPCR analysis was performed as in Figure 2d. Additional Indian ink staining is shown for a general comparison of the protein content of the cell lysates. **a** Immunoblot for LULL1, torsinA and α-tubulin. A quantification of torsinA band intensity in 3 independent lysates and immunoblots is shown at the bottom. Note the TorsinAp band, a putative stress-induced proteolytic cleavage product, appearing only in patient cell lysates.

b Additional qPCR analysis for LAP1 mRNA levels, performed as in Figure 2d, with a second primer set located upstream of the c.961C>T mutation. The first primer set was located downstream of the mutation at the exon 8-exon 9 splice junction. Since there are no splice junctions upstream of

the mutation, internal exon 1 sequences were used here. Despite this technical drawback, qPCR clearly shows a strong decrease of LAP1 mRNA levels in the two patient-derived cell lines. The results were normalized to β-tubulin and represent the means and s.d. of three independent experiments. **c** Additional immunoblot with an anti-lamin A/C antibody (compare to Figure 2c). **d** A longer exposure of the anti-LAP1 stained immunoblot shown at the top of Figure 2c. Red asterisks mark potential short translation or truncation products of LAP1.

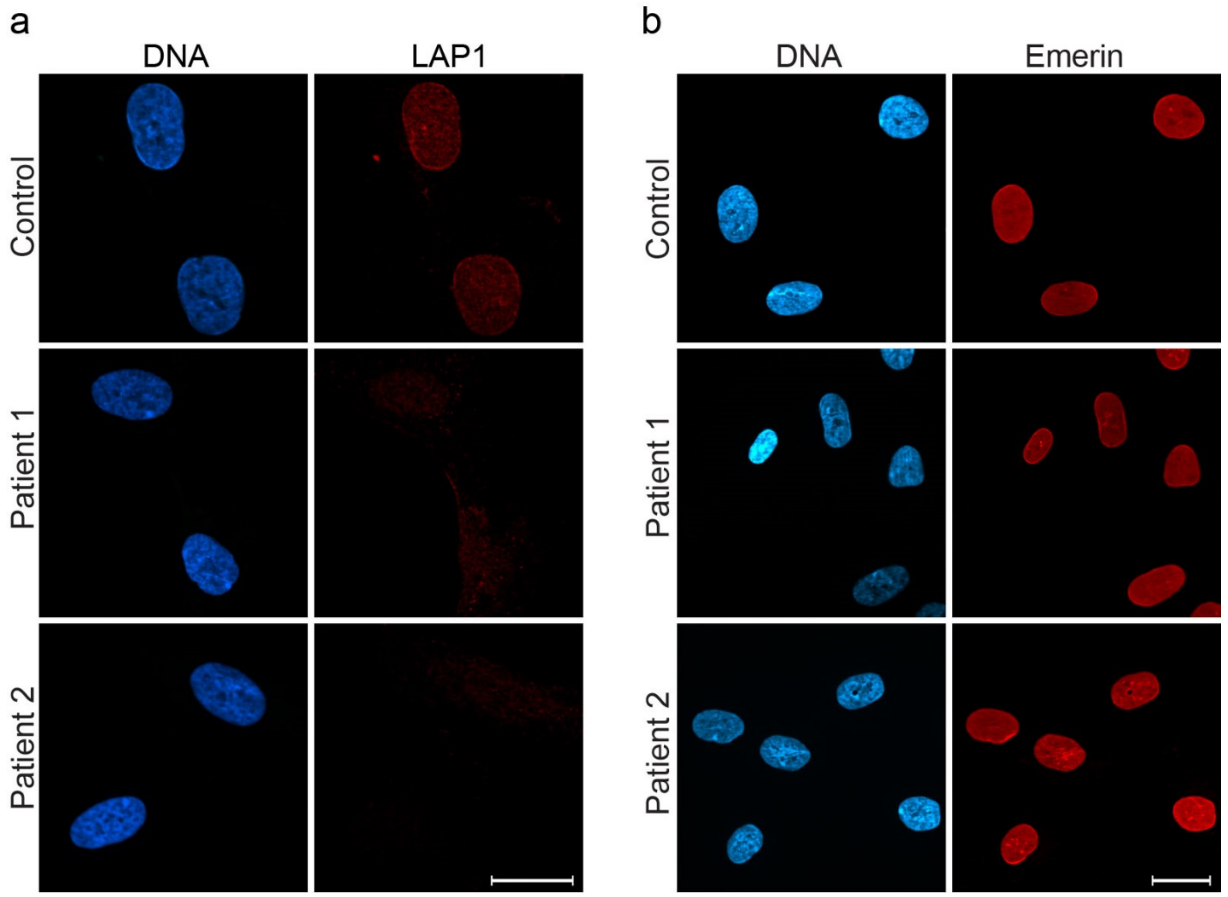

Supplementary Figure 3. Immunostaining for LAP1 and emerin in control and patient-derived fibroblasts. Widefield indirect immunofluorescence staining was performed as in Figure 3b. **a** Staining with a commercial anti-LAP1 antibody generated against a common region for the LAP1B and LAP1C isoforms. The typical appearance of a nuclear disk with pronounced rim staining was only evident in control cells and was missing in both of the patient-derived cell lines. **b** Staining with an anti-emerin antibody. Although immunoblot analysis of total cell lysates indicated an increase in emerin protein levels in patient cells (Figure 2C), we did not detect any difference in the immunofluorescence staining pattern between control and patient-derived fibroblasts (Patient 1: III-3; Patient 2: IV-4). Scale bars, 20 µm.

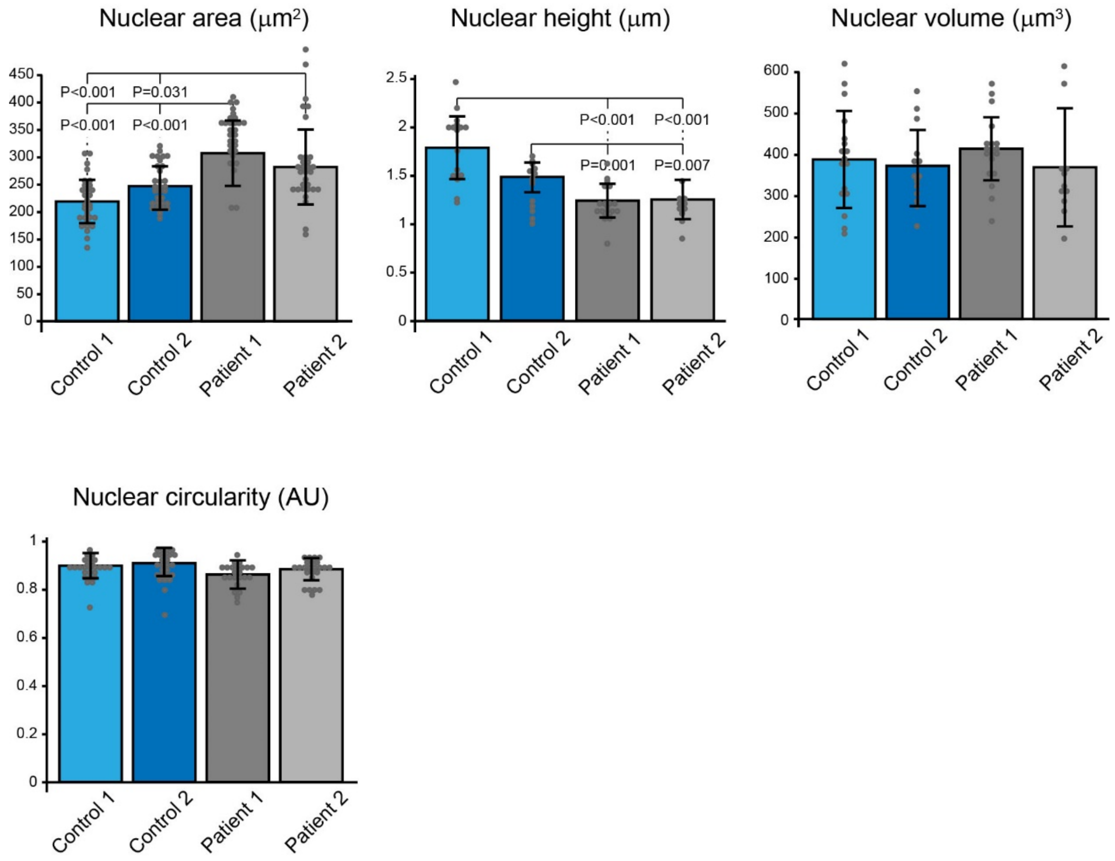

Supplementary Figure 4. Quantitative analysis of nuclear morphology parameters in control and patient-derived fibroblasts. Nuclei in two control and two patient-derived cell lines were stained by mAb414 and imaged by confocal microscopy as in Figure 4, including the acquisition of full Z-stacks of optical sections, taken with a 70 nm step size through each cell. Nuclear morphology parameters were measured using the ImageJ and Leica Application Suite X programs. Upper and lower tangential planes and a mid-section of mAb414 staining were identified for each nucleus. Nuclear height was defined as the distance between the upper and lower tangential planes. The nuclear perimeter was measured in ImageJ and nuclear area, volume and circularity were derived and calculated from these parameters as detailed in the Methods section. Statistically significant differences were only found for a lower mean height and larger mean area in patient-derived nuclei. Surprisingly, although some deformation of nuclear shape had been observed in patient nuclei, the overall calculated circularity was not affected. n=26 (Control 1), n=26 (Control 2), n=35 (Patient 1), n=24 (Patient 2); bars indicate SEM.

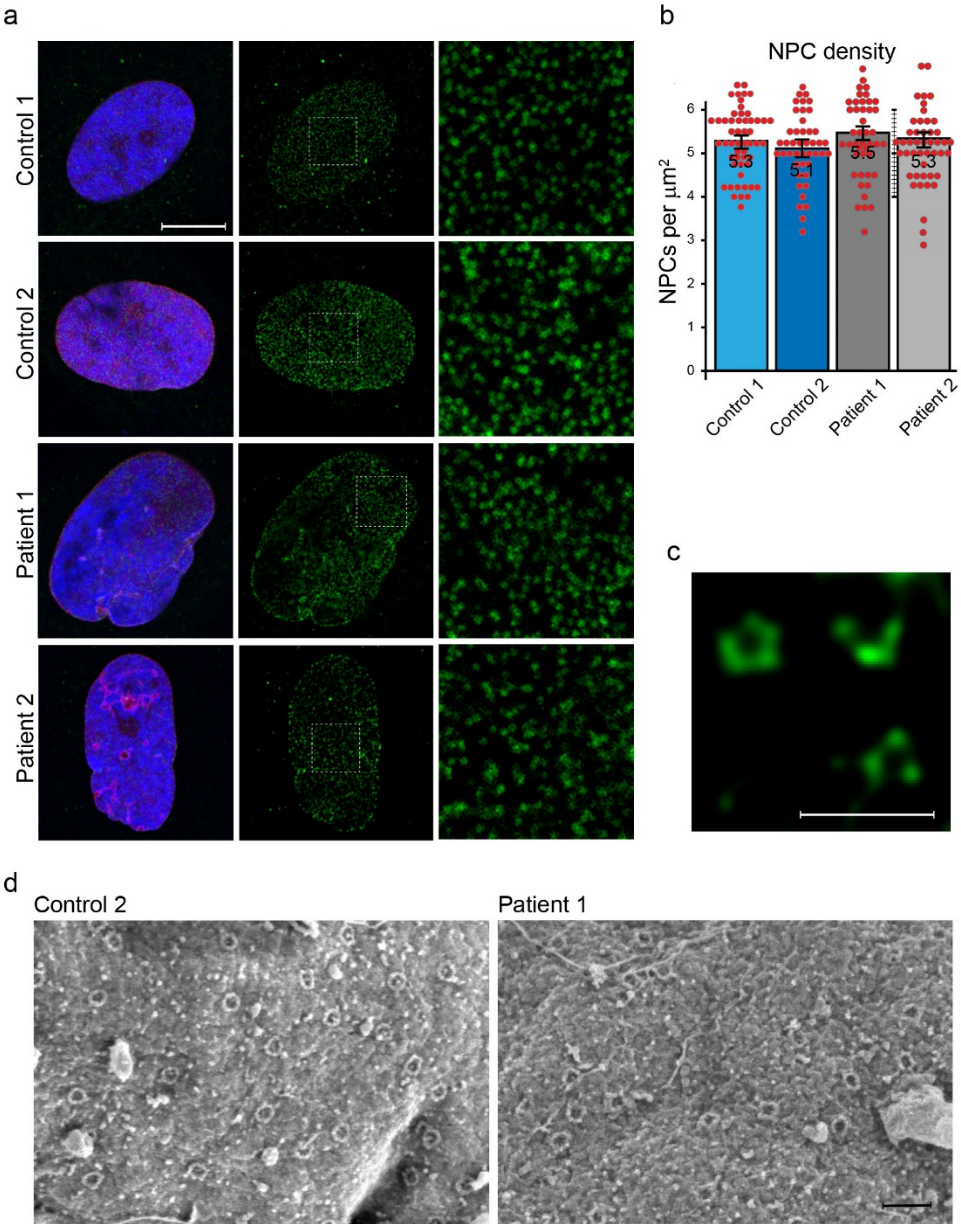

Supplementary Figure 5. Normal distribution and structure of nuclear pore complexes are observed in patient-derived nuclei. **a** Nuclei in two control and two patient-derived cell lines were stained by mAb414 and imaged by confocal microscopy as described in Supp. Figure 4. Upper tangential views are shown and boxed areas are enlarged in the right column, showing that the overall distribution of mAb414-staining *punctae* remains unchanged. Scale bar, 10 µm. **b** The estimated density of nuclear pore complexes based on mAb414-staining *punctae* was calculated for ≥3 randomly chosen areas in each nucleus for ≥20 cells in each category. **c** Enlarged panel of mAb414 staining in STEM mode, demonstrates the specificity of the antibody staining, showing individual nuclear pores with clear rotational symmetry. Scale bar, 200 nm. **d** Direct surface imaging of exposed nuclei from control and patient-derived fibroblasts by scanning electron microscopy. Two representative images are shown, demonstrating normal distribution and structural appearance of nuclear pore complexes. Scale bar, 200 nm.

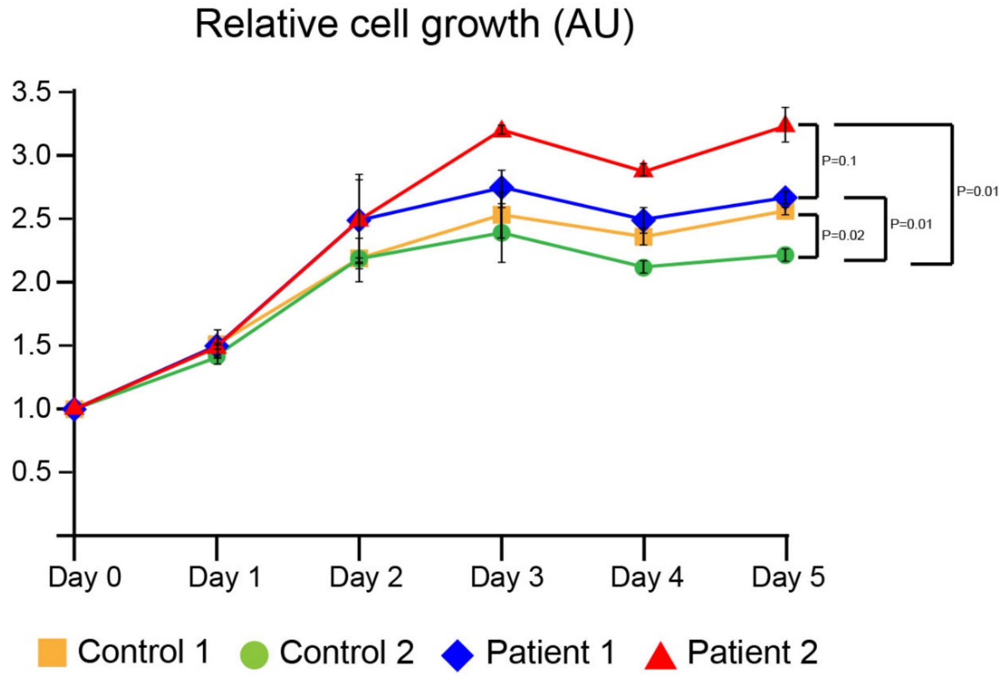

Supplementary Figure 6. Patient-derived primary fibroblasts show a slightly elevated proliferation rate compared to controls. Two control and two patient-derived primary fibroblast lines were grown under identical conditions in 96-well microplates and tested in a tetrazolium salt (XTT) colorimetric assay over a period of 5 days. The measured absorbance of formazan dye directly correlates to the number of metabolically active cells in the culture. Data are shown as mean ± SEM for two independent experiments performed in six replicates.

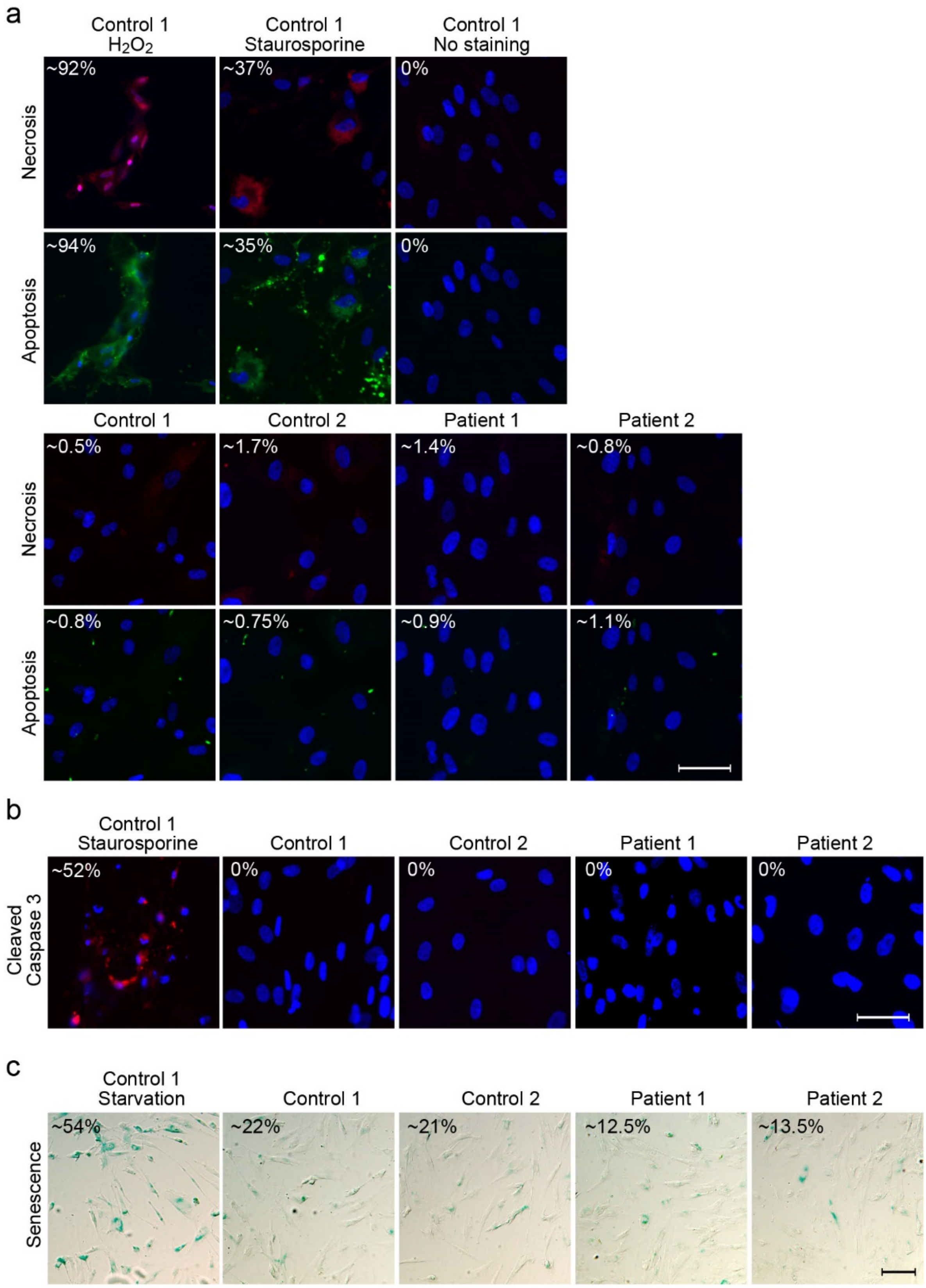

Supplementary Figure 7. A comparison of cell death and senescence markers between patient-derived and control fibroblasts. Two control and two patient-derived primary fibroblast lines were grown under identical conditions and analyzed by widefield microscopy. **a** Staining with fluorescent

markers for apoptotic (green, Apopxin Green indicator) and necrotic (red, membrane-impermeable 7-AAD) cell death markers. Top rows: positive (pre-treatment with 10 µM staurosporine or 2 mM H_2O_2) and negative (no staining) controls; bottom rows: untreated control and patient-derived cell lines. Representative fields are shown. Cells were visually scored according to positive control staining. Low staining levels were observed and no significant difference was found between control and patient-derived fibroblasts. n=200 for two independent experiments performed in duplicates. Scale bar, 50 µm. **b** Indirect immunofluorescence staining for anti-cleaved caspase-3, as an additional marker for apoptosis. Only the staurosporine positive control produced active caspase-3 staining. n=200 in two independent experiments performed in duplicates. Scale bar, 50 µm.
c Cellular senescence was assayed by staining for β-galactosidase activity at pH 6. Starvation-induced senescence served as a positive control (~54% of a control cell line stained positively). Representative fields are shown. Cells were visually scored according to positive control staining. A lower frequency of staining was consistently observed for all replicates of patient cells compared to the controls. n=250 for two independent experiments performed in duplicates. Scale bar, 100 µm.

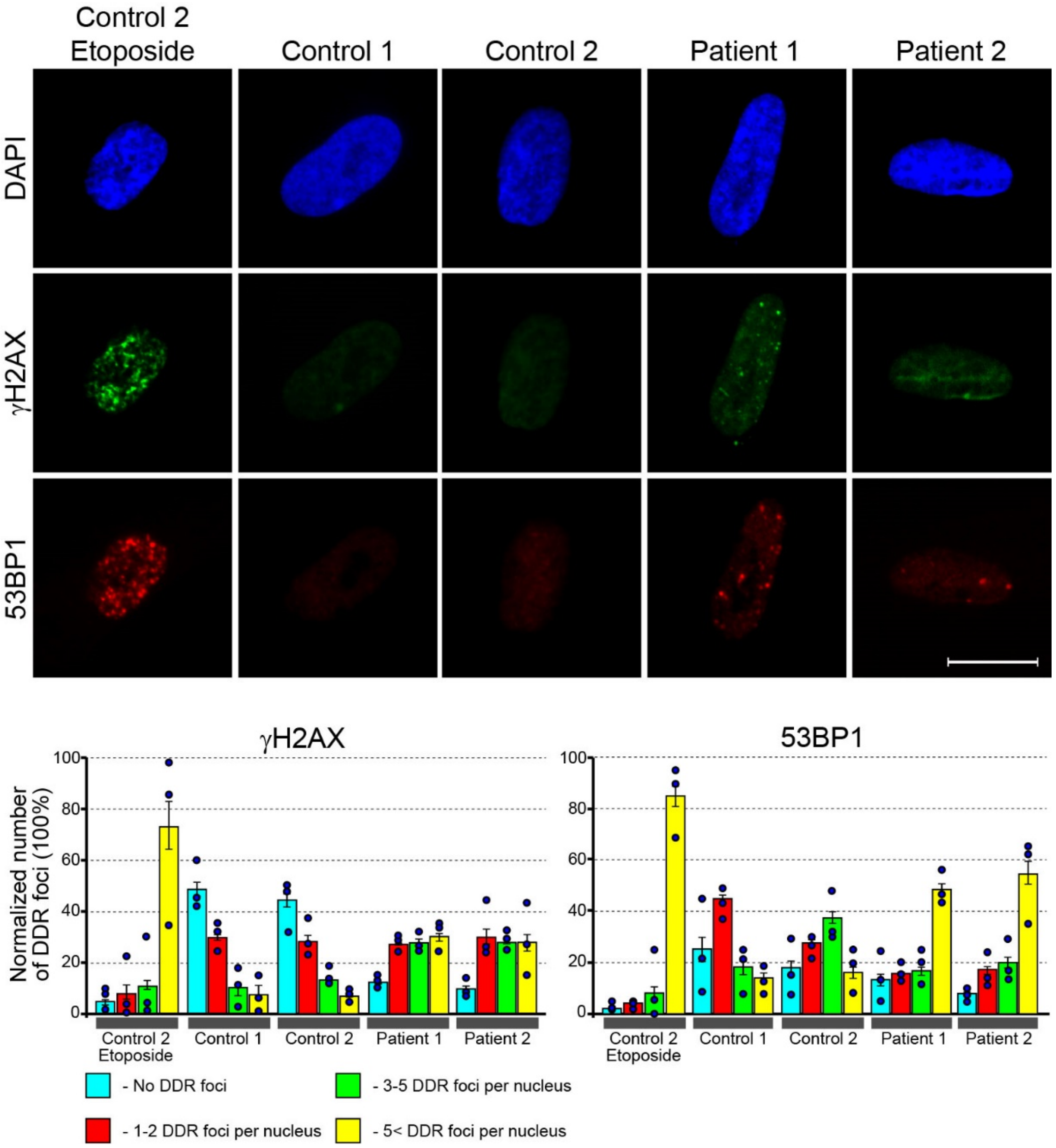

Supplementary Figure 8. A mild increase in staining for DNA damage response markers is observed in patient-derived fibroblasts. To assay for enhanced DNA damage and/or an impaired DNA damage response pathway, two control and two patient-derived primary fibroblast lines were grown under normal conditions and stained by indirect immunofluorescence using anti-γH2AX and anti-53BP1 antibodies. Pre-treatment with 30 µM etoposide served as a positive control producing a large number of stained foci for both markers in most nuclei (left column of images). Representative images of staining in untreated cells from control and patient-derived cell lines are shown in the next 4 columns of images. A significant difference between control and patient-derived cells emerged when categorized according to the number of foci per nucleus, as shown in the bar charts.
n≥50; data are shown as mean ± SEM for 3 independent experiments performed in duplicates. Scale bar, 20 µm.

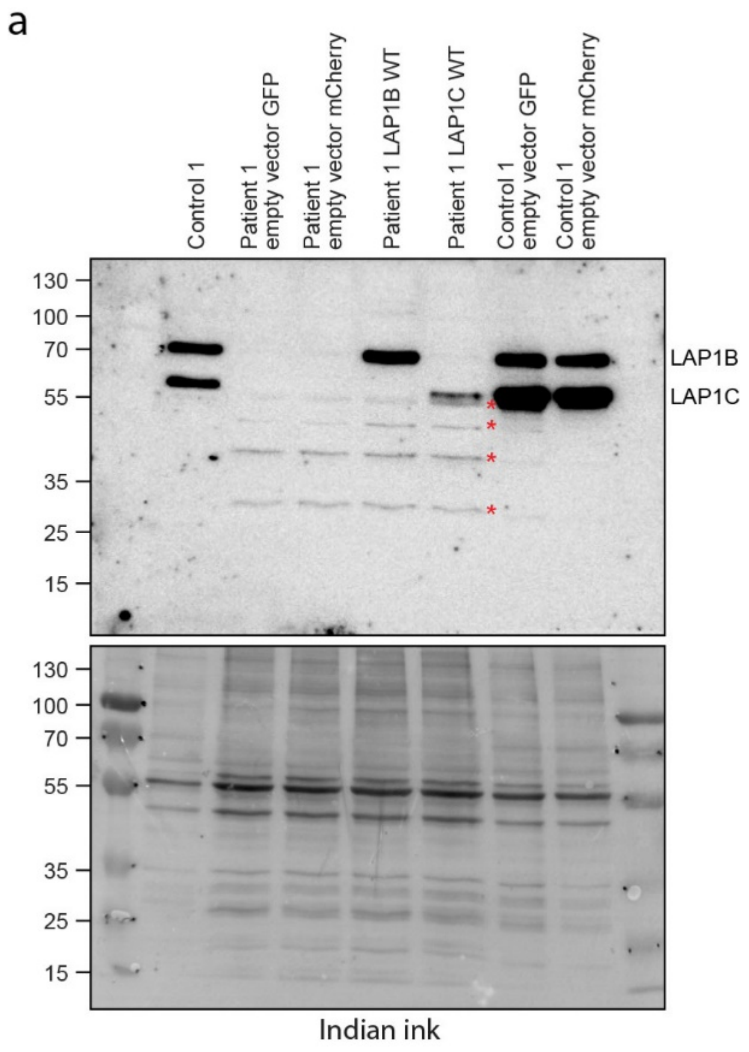

Supplementary Figure 9. Additional data for rescue experiments performed by stable transfection of LAP1-coding constructs. A comparison of LAP1B and LAP1C expression levels in different transduced cell lines and controls. Immunoblot analysis of total cell lysates was performed as described in Figure 2 and Supp. Figure 2. Note that stably transfected LAP1B is expressed in Patient 1 cells at a similar level to the normal isoform in untreated Control 1 fibroblasts. By contrast, the full length LAP1C isoform is only detected at a low level in transfected Patient 1 cells, along with shorter translation or truncation products (red asterisks; compare to Supp. Figure 2d).
