# Supplementary material for: Combined loss of LAP1B and LAP1C results in an early onset multisystemic nuclear envelopathy
Source: Nat Commun. 2019 Feb 5;10:605. doi: 10.1038/s41467-019-08493-7 (PMC6363790; doi:10.1038/s41467-019-08493-7)
Supplement: Supplementary file 3 — Description of Additional Supplementary Files [file 41467_2019_8493_MOESM3_ESM.pdf]

Supplementary Movie 1.

Time lapse microscopy of wound closing activity of control fibroblasts (well #D2) over a period of 126 hours in the IncuCyte ZOOM™ scratch wound assay.

Supplementary Movie 2.

Time lapse microscopy of wound closing activity of patient-derived fibroblasts (Patient 1: III-3; well #C4) over a period of 126 hours in the IncuCyte ZOOM™ scratch wound assay.

Supplementary Movie 3.

Time lapse microscopy of wound closing activity of patient-derived fibroblasts (Patient 2: IV-4; well #C8) over a period of 126 hours in the IncuCyte ZOOM™ scratch wound assay.
